# Circulating miR-34a and Bone Mineral Density of Brazilian Very-Old Adults

**DOI:** 10.1155/2020/3431828

**Published:** 2020-04-24

**Authors:** Otávio T. Nóbrega, Gilberto S. Morais-Junior, Nayara I. Viana, Sabrina T. Reis, Diego I. V. Perez, Wladimir M. Freitas, Andrei C. Sposito, Kátia R. M. Leite, Miguel Srougi

**Affiliations:** ^1^Federal University of Brasilia (UnB), Brasília, DF, Brazil; ^2^State University of São Paulo (USP), São Paulo, SP, Brazil; ^3^Universidad Santo Tomás, Puerto Mont, Chile; ^4^State University of Campinas (UNICAMP), Campinas, SP, Brazil

## Abstract

The human aging is marked by several body changes, including in bone mineral density (BMD). Research shows that microRNAs are important modulators of bone metabolism. The present research aims to analyze the whole blood concentration of 10 selected microRNAs (miRs) and their association with absolute and relative scores of BMD in specific osseous site of Brazilian very-old adults. Forty noninstitutionalized and apparently healthy, very old (≥80 years) outpatients were eligible for research. Anthropometry, biochemistry, and densitometry measurements were performed along with coronary artery calcification (CAC) scores and tested across total circulating levels of microRNAs. As expected, the relative BMD scores for the lumbosacral region (L1 to S5) and for the femoral head and neck observed in the sample denote weakened bone architecture, compatible with prevalent osteopenia and osteoporosis. In this context, one single significant association was found, and negatively implicated the miR-34a-5p with both absolute (*β* = −0.36, *P*=0.001 for BMD) and relative (*β* = −0.43, *P*=0.001 for T-score) densitometry indexes of the femoral head (adjusted to sex and physical activity practice), but not with the other sites. No difference in total blood concentrations of the miRs was found according to CAC scores. Our findings indicate greater circulating levels for miR-34a-5p among very-old adults who display the lowest scores of BMD, being a finding consistent with a modest contribution of the miR (along with co-variables) to the mineralization of that site. Attesting clinical relevance of our findings demands forthcoming studies.

## 1. Introduction

The human aging is marked by several body changes and greater risk for disorders as hormonal and metabolic imbalances as well as sarcopenia and osteoporosis. This last entity is characterized by progressive bone deterioration and a concomitant increase in risk for fractures among very-old men and women [1, 2]. Osteoporosis is mostly (but not solely) derived from loss of bone mineral density (BMD) and is increasingly potentiated by meno/andropausal-related hormonal changes, sedentary lifestyle, and other age-related conditions [3, 4].

BMD is maintained by the precise balance between formation and reabsorption of calcium in all bones, a balance accomplished by osteoclasts and osteoblasts [5]. Current research shows that microRNAs (miRs) are important modulators of bone metabolism [6, 7], with miRs as small non-protein-encoding RNA molecules containing 18 to 23 nucleotides in average, in one single-stranded chain [8, 9].

Being responsible for the posttranscriptional silencing of thousands of genes, mostly by the inhibition of protein translation, miRs are associated with virtually all biological process, including the regulation of the mineral content in osseous structures [6–10]. Since the onset of the bone mineralization process, several *in vitro* studies point out developmental roles for miRs [11–14], whereas experimental studies show differential *in situ* expression of these elements in osteoporotic bones [15–17]. By now, a body of evidence shows that a specific set of miRs (including miR-1, miR-92a, miR-126, miR-130a, miR-146a, miR-155, and miR-221, for instance) directly associate with the age-associated decline in BMD [18–22]. In this context, the miR-34a is suggested as a powerful suppressor of osteoclastogenesis and of calcium reabsorption in bones [23].

In this context, there is report of overexpression of the miR-21 and miR-100 in blood samples of patients with osteoporosis, in a manner that is proportional to scores of bone mineral density [24]. This evidence brings about a plausible role of miRs as potential biomarkers for decline of BMD and, if implicated in the osteoporosis pathophysiology, as eligible targets for preventive and therapeutic strategies of the condition. Thus, the present research has main objective to analyze the association of these 10 selected miRs with scores of bone mineral density of Brazilian very-old adults.

## 2. Materials and Methods

### 2.1. Subjects

All patients resided in the Brazilian Federal District, located in the Midwest Brazil. This cross-sectional report analyzed data from consecutive noninstitutionalized apparently healthy ambulatory patients, aged 80 years or more, participants in the ongoing Brazilian Study on Healthy Aging, a prospective study designed to identify markers for primary prevention of vascular events and other prevalent health outcomes in geriatrics settings [25, 26]. For this report, a subset that has never manifested bone fractures or recurrent falls was analyzed. Additional selection criteria were the absence of autoimmune disease (including rheumatic disorders), chronic or recurrent infections, prior or current neoplastic disease, or use of steroidal or nonsteroidal anti-inflammatory drugs in the past 30 days. Use of BMD-related drugs was investigated for each patient. The present report presents analyses of admission data of 40 subjects who fulfilled inclusion criteria. None were under any particular dietary regimen or were regular practitioners of physical exercises.

This study was approved by the institutional ethics committee and conducted following the Helsinki Declaration. Participation was voluntary, and written informed consent was obtained from each participant.

### 2.2. Anthropometric Characteristics

Anthropometric assessments were body mass index (BMI); weight (kg)/height (m^2^); and waist circumference (WC), according to the standards recommended by the World Health Organization [27].

### 2.3. Biochemical Analysis

At baseline, and after 12 h of overnight fasting, each participant underwent blood drawing for biochemical analysis and freezing at −80°C of serum and whole blood samples. For metabolic analysis, the following measures were performed using reagents from Roche Diagnostics (Mannheim, USA) and carried out by the same certified clinical laboratory according to routine clinical biochemistry, being expressed in standard units: blood glucose, glycated hemoglobin (HbA1c), total cholesterol and fractions, triglycerides, total serum protein, high sensitivity C-reactive protein, aspartate aminotransferase, alanine aminotransferase, alkaline phosphatase, gamma-glutamyl transferase, uric acid, and serum creatinine. Creatinine clearance was estimated using serum creatinine as described by Cockcroft and Gault [28].

Also, each individual underwent a detailed clinical examination. Type-2 diabetes was characterized by fasting blood glucose ≥126 mg/dl or use of insulin or oral antidiabetic drugs [29]. Hypertensive cases were defined following to the VII Brazilian Guidelines of Hypertension [30]. Metabolic syndrome was defined by the criteria of the National Cholesterol Education Program Adult Treatment Panel III [31]. Consumption of drugs to treat BMD-related conditions was investigated and recorded. Subjects were considered physically fit according to standards set by the World Health Organization for prescription of exercises for older adults [32].

### 2.4. Dual Energy X-Ray Absorptiometry Measurements

Participants underwent bone mineral density (BMD) assessments of the femoral neck, femoral head, and lumbarsacral (L1 to S5) regions with dual energy X-ray absorptiometry (DXA; (Lunar Prodigy Advance, GE Healthcare, USA) according to standard protocol to measure BMD (g/cm^2^). The difference between an individual's BMD and the mean BMD for a reference population was expressed in standard deviation term (T-score). Also, fat mass (kg) was incidentally measured during the procedure.

### 2.5. Cardiac Computed Tomography

Coronary artery calcification (CAC) was assessed by computed tomography, performed in a 64-slice scanner (Aquillion 64, Toshiba, Ottawara, Japan). Axial slices of 3 mm thickness with 3 mm table-feed were acquired at 70% of R-R interval with prospective electrocardiography triggering. CAC was defined as a minimum of 3 contiguous pixels with a peak Hounsfield unit density >130, being scored by a certified radiologist using the Agatston score to express its extent.

### 2.6. MicroRNA Selection

To select the set of microRNAs to be investigated, we have searched the DIANA Tools (TarBase v7.0) [33] for miRs validated as interacting with target genes encoding key noncollagenous, mineralization-associated proteins of the bone matrix, as described by Javed et al. [34]. A manual search was performed by entering Ensembl code individually, with each human(hsa)-miR being selected for analyses when the database reported an association (positive result) and the source tissue specified as bone. From this strategy, 10 miRs were rendered eligible for analyses, as follows: miR-1-3p, miR-21-5p, miR-34a-5p, miR-92a-3p, miR-100-5p, miR-126-3p, miR-130a-3p, miR-146a-5p, miR-155-5p, and miR-221-3p.

### 2.7. MicroRNA Analysis

All blood samples were obtained by puncture of the antecubital vein with 1 ml aliquots immediately frozen and stored at −80°C until further processing. Before starting the extraction procedure, samples were thawed on ice. Total RNA (including small RNAs) was purified from 700 µl blood samples using the miRVana™ PARIS™ kit (Thermo Fisher Scientific, Wilmington, DE, USA) adapted by adding multiple acid-phenol : chloroform extraction steps to produce clearer aqueous phases. Concentration and optical quality of samples were determined using the NanoDrop Lite. Reactions of cDNA synthesis for each miR were performed with the TaqMan® microRNA Reverse Transcription Kit (Thermo Fisher Scientific) and the appropriate TaqMan® RT primer, with 30 min incubations at 16°C and then at 42°C, terminated at 85°C for 5 min. The quantitative real-time PCR (qPCR) step was performed in 10 *μ*l reaction mixtures using a TaqMan Universal PCR Master Mix (Thermo Fisher Scientific) with specific TaqMan® assays (primers and probes) and the ABI 7500 equipment (Applied Biosystems, Foster City, CA, USA), following the manufacturer's instructions. RNU43 was used as endogenous control to normalize Ct values obtained for each target miR, with expression compared by means of the 2^−ΔCt^ method [35].

### 2.8. Statistical Analysis

To address the aim of evaluating the occurrence and strength of the association between whole blood, circulating levels of miRs, and scores (continuous and categorical) of anthropometric, clinical, and biochemical traits of potential confounding effect in the main model, our statistical analyses started at obtaining correlation coefficients between these variables. For that, normal distribution of all variables was assessed using the Kolmogorov–Smirnov test.

The association between continuous, normally distributed variables was evaluated using Pearson's correlation test, whereas the involvement of a least one categorical or nonnormally distributed continuous variable was dealt using a linear regression model. For these latter analyses, absence or presence of a given feature was represented by 0 or 1, respectively. When a variable was nonnormally distributed, a logarithmic transformation was applied. Whenever an interaction was noticed, regression analyses were run using adjustment for the confounding variable(s) or condition(s). For these analyses, a *P* value (two-tailed) was rendered significant following Bonferroni's principle of correction for multiple comparisons whenever a given trait is tested across *k* independent variables (e.g., if *k* = 10 tests, *α* = 0.005).

Also, Student's *t*-test was used to verify if miRs concentrations varied across the subjects grouped according to the median scores of CAC and BMD in the sample, with adjustment to covariates when appropriate. Finally, linear multivariate regression analysis, the stepwise method, was performed to assess at which extent the whole blood miRs concentrations explained the variability in the calcification indexes. All analyses were performed with the Statistical Package for the Social Sciences (SPSS) for Windows (version 17.0).

## 3. Results

After applying the exclusion criteria, 40 very-old subjects (men and women) were eligible for research. The common clinical, anthropometric, and metabolic features of the subjects investigated are summarized in Table 1. Most notably, average relative bone mineral density scores for the lumbosacral and the femoral head and neck were expressed in negative values, denoting weakened bone architecture among the subjects, compatible with the expected prevalence of osteopenia and osteoporosis.

Table 2 presents correlation and regression analyses of circulating levels of miRs with common clinical, anthropometric, and metabolic features of the very-old adults investigated. It became evident that the overall serum levels of miRs investigated vary neither according to the common characteristics as age, sex, or body fat nor across conditions as type-2 diabetes and hypertension (among other traits) presented by the volunteers. Complementarily, average miRs levels were also compared using the *t*-test across groups of dichotomic variables (sex, physical activity, T2DM, hypertension, and metabolic syndrome), and no difference was found (not shown). On the other hand, similar analyses found that average scores of BMD at all osseous sites varied according to sex (*P* < 0.001) and physical activity practice (*P*=0.005) (not shown in Tables), with these traits being included as covariables in the forthcoming inferential analyses.

The linear regression analyses of circulating levels of miRs with the mineral content in the coronary and the osseous territories are summarized in Table 3. According to the significance threshold adopted herein, one single relevant relationship was found and negatively implicated the miR-34a with both absolute (*β* = −0.36, *P*=0.001 for BMD) and relative (*β* = −0.43, *P*=0.001 for T-score) mineral density scores in the femoral head. Similar associations were observed neither for the lumbosacral region nor the femoral neck.

**Figure 1:**
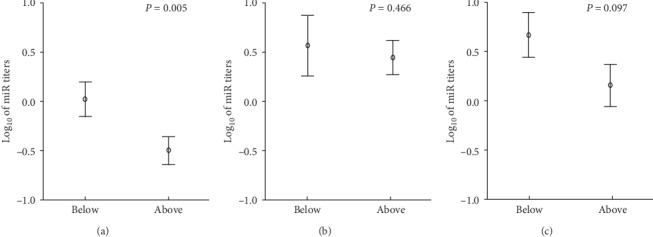
Comparison of log-transformed circulating levels of miR-34a (a), miR-126 (b), and miR-146a (c) across individuals grouped according to the median (0.82 g/cm^2^) bone mineral density in the femoral head. Significance was verified by MANOVA with adjustment to sex and physical activity practicing. Vertical bars represent intervals of one standard error.

Figure 1 presents comparison of log-transformed levels of all three microRNAs with greater trends for an association (from the prior analyses) with the femoral head BMD (miR-34a, miR-126 and miR-146) across individuals grouped according to the median value (0.82 g/cm^2^) of bone mineral density in the femoral head. Results reassure that a significant difference could only be devised for miR-34a, with a greater concentration of circulating level for miR-34a among carriers of lower BMD. No difference was found in total blood concentrations of any of the miR tested across the sample split according to median CAC scores, with or without adjustments.

## 4. Discussion

A baseline finding in our setting consisted in mean negative values for relative scores of BMD on all osseous sites investigated (lumbosacral and femoral head and neck) in the sample of very-old adults. Regardless of this expected scenario of an important age-related decrease of BMD, a remarkable and specific negative association of miR-34a with the bone density scores of the femoral head could be devised. Also, as expected, mineral density scores were significantly higher for men than for women at all osseous sites investigated (*P* < 0.001). But, our results seem not biased by admitting both sexes since the male/female ratio did not vary across the sample slip according to median scores of BMD (*χ*^2^ = 2.157; *P*=0.608; for femoral head as example) or CAC (*χ*^2^ = 2.837; *P*=0.387). Thus, we suggest that the miR-34a (among all miRs studied) exhibited a consistent relationship with the mineralization status of the femoral bone, so to be detected out of a sampling of the peripheral bloodstream. Stepwise multivariate regression showed that the miR-34a levels along with sex and physical activity accounted for roughly 58% of the variance in the mineral density of the femoral head.

It has been demonstrated that an *in vitro* inhibition of miR-34a by means of antisense-miR was responsible for an increased differentiation of osteoblasts and, consequently, an increase in cultured bone matrix formation [11, 23]. On the other hand, overexpression of miR-34a caused an inverse effect. Thereby, research elsewhere already suggests that *in situ* expression of mirR-34a it is negatively correlated to BMD, rendering levels particularly high of this element as a plausible biomarker for osteoporosis [15]. Nonetheless, the actual physiological mechanism(s) on how miR-34a acts to suppress matrix mineralization is yet to be fully described.

In this respect, there is evidence that the expression of transforming growth factor-*β*-induced factor 2 (Tgif2) during *in vitro* formation and differentiation of both animal and human osteoclasts is targeted by miR34a [11]. Tgif2 is soundly acknowledged as a proosteoclastogenic factor and miR34a inhibits its expression, consequently leading to decreased calcium reabsorption and bone matrix renewal [23]. These results are consistent with our findings on elderly with lower BMD showing more pronounced total circulating levels of miR-34a.

Despite the homogeneity of our subjects on what concerns age strata, clinical conditions, and biochemical profile, our investigation has limitations greatly related to factors inherent to a developing country as Brazil and that were not investigated herein. The remarkable interethnic variation owing to genetic admixture is certainly one of these factors [36]. Also, diversity in functional capacity and in food intake cannot be ruled out as confounding factors. Besides all, the most remarkable limitation may be represented by the difficulty of relating findings in a peripheral blood sample with pathophysiological processes in specific tissues/organs. In this respect, one should note that bone-derived exosomes are released into the extracellular environment [37], and it is plausible that at least part of the microRNA content in the whole blood may reflect concentrations (and imbalances) existing elsewhere, including the bone millieu.

So far, the authors are unaware of other studies that have correlated total circulating levels of microRNAs in the blood with bone mineral density of very-old women and men. Our aged human model suggests that total concentration of miR-34a explains at least in part the complex phenotype of bone structure. However, attesting clinical relevance of the finding presented herein still depends on forthcoming studies, so as to assess its diagnostic value (if any) for future clinical practice.

## Figures and Tables

**Table 1:** Clinical, anthropometric, and metabolic features of the 40 very-old subjects investigated.

Variable	Value
Women (%)	70.0
Age (years)	84.2 ± 4.5
BMI (kg.m^−2^)	26.6 ± 3.4
WC (cm)	95.6 ± 9.8
Body fat (%)	17.2 ± 4.1
Glycated hemoglobin (%)	5.9 ± 0.5
Fasting glucose (mg.dl^−1^)	96.3 ± 11.7
Type-2 diabetes (%)	22.5
SBP (mm Hg)	143.9 ± 18.9
DBP (mm Hg)	72.7 ± 11.2
Hypertension (%)	75.0
Triglycerides (mg·dl^−1^)	124.3 ± 59.8
Total cholesterol (mg·dl^−1^)	190.9 ± 42.7
HDL-c (mg·dl^−1^)	52.4 ± 13.6
Metabolic syndrome (%)	50.0
C-reactive protein (mg·dl^−1^)	1.4 (1.0, 2.4)
Agatston score	20.5 (0.2, 907.0)
Lumbosacral BMD (g·cm^−2^)	0.89 ± 0.15
Lumbosacral T-score	−1.53 ± 1.23
Femural head BMD (g·cm^−2^)	0.84 ± 0.14
Femural head T-score	−1.00 ± 0.93
Femural neck BMD (g·cm^−2^)	0.70 ± 0.12
Femural neck T-score	−1.45 ± 0.99
Use of BMD-related drugs (%)	47.5
Exercise practitioners (%)	50.0
Aspartate aminotransferase (U/l)	21.0 ± 6.0
Alanine aminotransferase (U/l)	15.5 ± 5.4
Alkaline phosphatase (U/l)	71.1 ± 23.0
Gamma-glutamyl transferase (U/l)	21.9 ± 11.5
Uric acid (mg/dl)	5.23 ± 1.26
Total serum protein (g.dl^−1^)	7.36 ± 0.46
Serum creatinine (mg.dl^−1^)	0.99 ± 0.33
Creatinine clearance (ml.min^−1^)	47.9 ± 15.2

Data expressed as mean ± standard deviation, proportion or median (and interquartile range). BMI = body mass index. WC = waist circumference. SBP = systolic blood pressure. DBP = diastolic blood pressure. HDL-c = high-density lipoprotein cholesterol. BMD = bone mineral density.

**Table 2:** Correlational and linear regression analyses of circulating levels of miRNAs with common clinical, anthropometric, and metabolic features of the very-old subjects investigated.

Traits	Log-transformed levels of microRNAs									
	miR1	miR21	miR34a	miR92a	miR100	miR126	miR130a	miR146a	miR155	miR221
Age	−0.02; 0.922	−0.22; 0.166	0.07; 0.663	−0.16; 0.311	−0.13; 0.412	0.05; 0.752	0.06; 0.698	−0.01; 0.937	−0.20; 0.208	−0.01; 0.944
Sex^§^	0.29; 0.059	−0.21; 0.631	0.16; 0.603	0.06; 0.945	0.11; 0.763	0.26; 0.408	0.28; 0.642	−0.21; 0.631	0.06; 0.926	−0.57; 0.260
Body fat	0.14; 0.385	0.18; 0.256	−0.07; 0.687	0.15; 0.363	0.16; 0.309	−0.15; 0.366	0.21; 0.185	0.04; 0.830	0.19; 0.244	0.23; 0.147
T2DM^§^	0.43; 0.262	−0.41; 0.326	−0.56; 0.070	0.54; 0.556	0.03; 0.935	−0.20; 0.503	−0.29; 0.623	0.96; 0.027	−0.53; 0.424	0.06; 0.907
Hypertension^§^	0.16; 0.516	−0.08; 0.771	−0.01; 0.954	−0.05; 0.933	0.26; 0.278	−0.17; 0.385	0.08; 0.840	0.06; 0.830	0.63; 0.145	−0.16; 0.600
Physical activity^§^	0.13; 0.715	0.12; 0.749	−0.10; 0.727	−0.74; 0.382	0.41; 0.223	0.08; 0.776	−0.82; 0.138	0.61; 0.117	0.66; 0.284	0.10; 0.819
Metabolic syndrome^§^	−0.14; 0.642	−0.17; 0.613	−0.07; 0.767	1.41; 0.069	−0.04; 0.907	−0.24; 0.324	0.38; 0.434	−0.04; 0.907	−0.42; 0.439	−0.20; 0.613
Log C-RP	0.05; 0.770	−0.06; 0.710	0.30; 0.057	0.09; 0.581	0.11; 0.509	−0.02; 0.919	0.16; 0.320	0.13; 0.432	−0.06; 0.711	0.09; 0.577
Serum creatinine	−0.07; 0.659	−0.04; 0.826	−0.08; 0.642	−0.04; 0.815	−0.22; 0.181	−0.01; 0.992	0.05; 0.771	0.02; 0.896	−0.01; 0.940	−0.02; 0.886
Creatinine clearance	−0.01; 0.983	0.01; 0.978	0.00; 0.998	0.02; 0.902	0.16; 0.315	−0.06; 0.734	−0.13; 0.436	−0.12; 0.474	0.02; 0.919	−0.05; 0.764

The Pearson correlation test (two continuous) or a Linear Regression Model^§^ (one continuous and one categorical) were used to test relationships between variables. Data are expressed as correlation index and significance level (*r*; *P*) or as beta coefficient and significance level (*β*; *P*) for correlational and regression analyses, respectively. T2DM = type 2 diabetes mellitus. C-RP = C-reactive protein. Log = logarithmically transformed.

**Table 3:** Linear regression analyses of circulating levels of microRNAs with mineral content in coronary and bone territories of the very-old subjects investigated.

Log-transformed levels of microRNAs	Log Agatston score	BMD^§^			T scores^§^		
		LS	FH	FN	LS	FH	FN
miR-1-3p	0.26; 0.320	−0.17; 0.247	−0.16; 0.193	−0.07; 0.658	−0.19; 0.237	−0.21; 0.143	−0.09; 0.565
miR-21-5p	−0.06; 0.858	0.09; 0.541	−0.12; 0.334	−0.02; 0.868	0.08; 0.604	−0.16; 0.261	0.02; 0.926
miR-34a-5p	−0.09; 0.780	−0.05; 0.735	−0.36; 0.001	−0.22; 0.130	−0.05; 0.745	−0.43; 0.001	−0.24; 0.131
miR-92a-3p	−0.42; 0.923	0.05; 0.734	−0.13; 0.294	−0.08; 0.583	0.03; 0.829	−0.17; 0.228	−0.05; 0.757
miR-100-5p	−0.04; 0.867	−0.04; 0.793	0.20; 0.092	0.26; 0.073	−0.06; 0.694	0.23; 0.093	0.25; 0.109
miR-126-3p	0.44; 0.147	0.05; 0.753	−0.27; 0.022	−0.15; 0.294	0.05; 0.771	−0.31; 0.022	−0.13; 0.409
miR-130a-3p	−0.81; 0.208	0.09; 0.545	−0.21; 0.078	−0.10; 0.505	0.10; 0.548	−0.26; 0.059	−0.07; 0.644
miR-146a-5p	0.13; 0.764	−0.01; 0.954	−0.25; 0.032	−0.16; 0.270	−0.01; 0.956	−0.30; 0.027	−0.13; 0.393
miR-155-5p	0.09; 0.787	0.14; 0.328	−0.14; 0.248	−0.17; 0.232	0.14; 0.387	−0.18; 0.189	−0.12; 0.449
miR-221-3p	1.00; 0.087	0.05; 0.735	−0.22; 0.062	−0.07; 0.618	0.05; 0.769	−0.27; 0.045	−0.06; 0.700

A Linear Regression Model was used, with adjustment^§^ to sex and to physical activity practicing. Data are expressed as beta coefficient and significance level (*β*; *P*). BMD = bone mineral density; LS–lumbarsacral; FH = femural Head; FN = femural Neck; Log = logarithmically transformed.

## Data Availability

Data can be made available upon reasonable request.
